# Plant Single-Cell Metabolomics—Challenges and Perspectives

**DOI:** 10.3390/ijms21238987

**Published:** 2020-11-26

**Authors:** Leonardo Perez de Souza, Monica Borghi, Alisdair Fernie

**Affiliations:** 1 Max Planck Institute of Molecular Plant Physiology, Am Müehlenberg 1, Golm, 14476 Potsdam, Germany; 2 Department of Biology, Utah State University, 1435 Old Main Hill, Logan, UT 84322, USA; monica.borghi@usu.edu

**Keywords:** cell type specific metabolism, metabolomics, single-cell, mass spectrometry imaging

## Abstract

Omics approaches for investigating biological systems were introduced in the mid-1990s and quickly consolidated to become a fundamental pillar of modern biology. The idea of measuring the whole complement of genes, transcripts, proteins, and metabolites has since become widespread and routinely adopted in the pursuit of an infinity of scientific questions. Incremental improvements over technical aspects such as sampling, sensitivity, cost, and throughput pushed even further the boundaries of what these techniques can achieve. In this context, single-cell genomics and transcriptomics quickly became a well-established tool to answer fundamental questions challenging to assess at a whole tissue level. Following a similar trend as the original development of these techniques, proteomics alternatives for single-cell exploration have become more accessible and reliable, whilst metabolomics lag behind the rest. This review summarizes state-of-the-art technologies for spatially resolved metabolomics analysis, as well as the challenges hindering the achievement of sensu stricto metabolome coverage at the single-cell level. Furthermore, we discuss several essential contributions to understanding plant single-cell metabolism, finishing with our opinion on near-future developments and relevant scientific questions that will hopefully be tackled by incorporating these new exciting technologies.

## 1. Introduction

**Figure 1:**
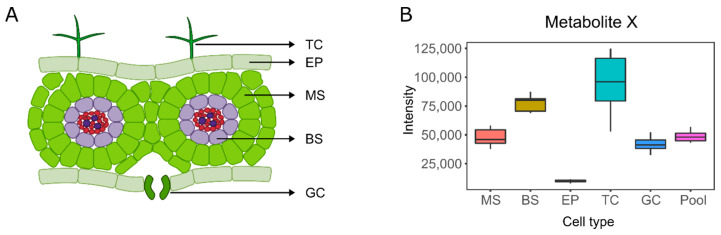
The effect of cell heterogeneity, exemplified by different cell types in a leaf, in metabolomics analysis. (**A**) Diagram of a leaf cross-section depicting typical cell types. (**B**) Hypothetical values for a metabolite X differentially accumulated in multiple cell types highlighting the averaging effect of pooling cells together in a traditional metabolomics experiment. Trichomes (TC), epidermal cells (EP), mesophyll cells (MS), bundle sheath cells (BS), and guard cells (GC).

The advent of genomics immediately followed by similar conceptual frameworks to investigate transcriptomes, proteomes, and metabolomes represented a paradigm shift in biological systems investigation. The appealing idea of holistically assessing such systems has translated into rapid developments for systems biology. Researchers can now investigate multiple processes simultaneously, revealing essential mechanisms involved in regulating development and responses to the environment. For practical reasons, such techniques have been mainly applied to bulk samples consisting of a large number of cells for which results correspond to populations’ averages (Figure 1). In such experiments, the stochasticity of biological processes leading to cell heterogeneity is often considered not to be biologically relevant. Indeed, this is often the case, and for many applications such as characterizing mutants of central metabolic pathways [1,2] and identifying genes involved in the production of specialized metabolites [3,4,5], the use of averages is undoubtedly suitable.

However, cell heterogeneity has been shown to play important biological roles in many situations for which averaging would mask relevant mechanistic insights [6]. In plants, several works highlighted the importance of cell-specific metabolism in regulating essential physiological processes such as the metabolism of the shoot apical meristem [7], the regulation of stomatal closure by guard cells and subsidiary cells [8,9], C4 metabolism [10,11,12], and the evolution of specialized metabolism [13]. However, most of these studies involve cell-specific labor-intensive protocols for cell isolation or reporter lines targeting few metabolites. True metabolomics at the cellular level remains a daunting task due to innumerable challenges in measuring metabolites.

## 2. Technical Challenges

Current coverage of the metabolome still lags far behind genomics, transcriptomics, and proteomics because of the technical limitations imposed by the nature of metabolites. DNA, RNA, and proteins exhibit high regularity as they are constituted by a set of repeating unities, namely nucleotides and amino acids. As a consequence, these classes of molecules have characteristic physicochemical properties that are similar between them. Metabolites, on the other hand, exhibit much broader physicochemical diversity hindering their global analysis by a single technique. The broadest coverages of the metabolome achievable by date rely heavily upon the high sensitivity of mass spectrometry techniques hyphenated to efficient separation provided by gas and liquid chromatography. Although current technological advances provide considerable resolution in benchmark equipment such as orbitraps and quadrupole time-of-flight mass spectrometers (QTOFs), the combination of these two techniques is still essential for overcoming matrix effects providing maximum metabolome coverage.

Following the trend, metabolomics once again lags its predecessors in the pursuit of single-cell systems biology. Single-cell genomics and transcriptomics saw rapid popularization in the last years [14], followed more recently by proteomics [15,16,17]. Here, in addition to the aforementioned technical hurdles, sensitivity also imposes a challenge for single-cell metabolomics. DNA and RNA analysis presents a significant technical advantage as the genetic material can be amplified, yielding considerably more sensitive detection over proteins and metabolites. Recent developments in proteomics have explored alternatives such as fluorescent tags providing a comprehensive increase in sensitivity. On the other hand, metabolites cannot be amplified, and their broad dynamic range of concentrations has a considerable impact on the observable metabolome. Furthermore, improving detection through derivatization reactions is also complicated by their wide chemical diversity and a higher propensity to structural modifications compared to bulkier proteins. Moreover, the minute concentrations and volumes of material represent an issue for using classical platforms relying on chromatographic separation.

**Figure 2:**
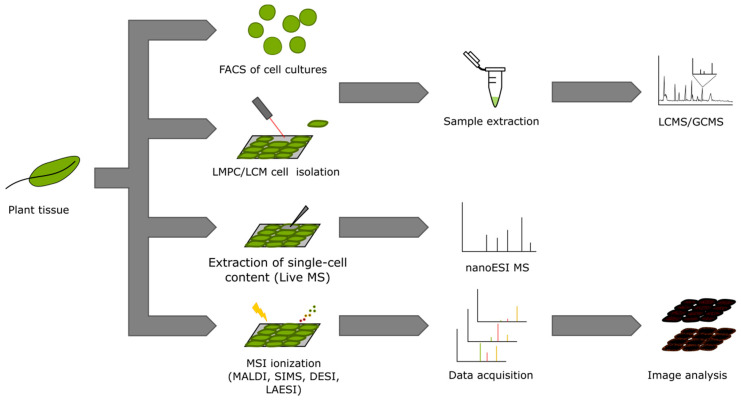
Overview of experimental steps and data structure from the different approaches for cell-specific metabolomics.

We can classify attempts to achieve cellular resolution metabolomics in three main groups: those that attempt at isolating enough material of a specific cell type to perform the analysis on platforms used for regular metabolomics, which we will refer to as single-cell-type metabolomics as coined by Reference [18]; those based on micromanipulation of single cells [19,20]; and those based on mass spectrometry imaging (MSI) [21,22]. In the next sections, we briefly summarize some of the main vantages and disadvantages of the different approaches (Figure 2).

## 3. Single-Cell and Single-Cell-Type Metabolomics

In an ideal scenario, direct extraction of the inner content of a cell, or the cell as a whole, followed by metabolite profiling, represents the optimal procedure for preserving the natural cellular environment and assessing individual cellular heterogeneity across an organism. However, mass spectrometry performed within such low volumes and concentrations is generally limited to detecting only a small set of compounds. Moreover, performing chromatography with such material is even more challenging, and most platforms skip this method altogether. The lack of chromatographic separation results in increased matrix effects such as ion suppression, simply put, the signal reduction due to ionization interference between species simultaneously reaching the ionization source, therefore, negatively affecting the detection of most analytes. One of the few platforms established for such analysis, “Live-MS” performs single-cell metabolite profiling by sucking out the cell content under video-microscopy observations with the help of a metal-coated microcapillary such as a nanospray tip. The sample is further transferred into a mass spectrometer via a nano-electrospray ionization plume [19,23].

A more viable alternative from the analytical point of view is to sample many specific cells before the metabolomics experiment in single-cell-type experiments [18]. The main advantage is the possibility of using traditional LC/GC-MS-based platforms providing high throughput, optimal sensitivity, and coverage due to the chromatographic separation. The main limitations are imposed by the chosen cell sampling technique [24,25,26]. Some particularly exciting works include applications of laser microdissection (LMD)-based techniques such as laser microdissection and pressure catapulting (LMPC) and laser capture microdissection (LCM) [18], as well as fluorescence-activated cell sorting (FACS) [27].

LMD-based techniques are a great option as they preserve contextual information from spatial cell distribution. However, they are significantly limited in terms of throughput. LMD is a labor-intensive technique requiring an experienced operator to harvest the cells [18]. FACS, on the other hand, provides a high throughput alternative to isolating specific cells. However, the necessity to obtain single-cell suspensions is far from trivial, considerably affecting the metabolome [28]. Moreover, the inherent introduction of perturbations due to cell manipulation by all these techniques is particularly troublesome when considering the rapid changes of the metabolome with the turnover time of some metabolites being fractions of a second [29]. Despite these limitations, the recent improvement in data processing capacity and machine learning algorithms brings exciting advances to fill some of these gaps. A great example has recently been shown using image analysis algorithms, machine-learning, and high-throughput microscopy to recognize individual cells in suspensions or tissue and automatically guide extraction through LCM or micromanipulation in the so-called computer-assisted microscopy isolation (CAMI) [30]. Similarly, exciting improvements have also been developed for FACS [31]. However, the issues related to obtaining cell suspensions for this technique are likely hard to overcome and particularly challenging for plant sciences, as discussed below.

## 4. Mass Spectrometry Imaging (MSI)

**Figure 3:**
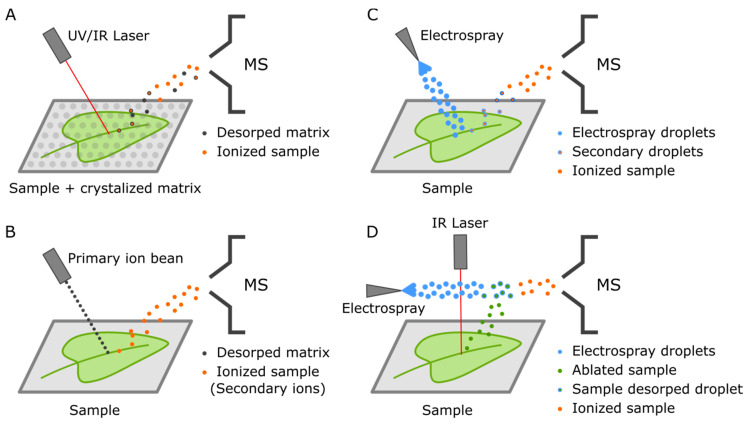
Schematic representation of the different ionization strategies used for mass spectrometry imaging (MSI). (**A**) MALDI, (**B**) secondary ion mass spectrometry (SIMS), (**C**) desorption electrospray ionization (DESI), (**D**) laser-ablation electrospray ionization (LAESI).

MSI is a general term encompassing multiple technologies capable of providing spatially resolved ionization of samples for mass spectrometry-based metabolite profiling [21,22]. The multiple techniques essentially provide different tradeoffs related to sample preparation, the lateral resolution of the ionization spot, degree of fragmentation, and ionization range (*m*/*z*). We briefly describe here some of the most common ionization platforms that we believe cover an attractive complementary space of features, namely matrix-assisted laser desorption/ionization (MALDI) [32,33], secondary ion mass spectrometry (SIMS) [34], desorption electrospray ionization (DESI) [35,36], and laser-ablation electrospray ionization (LAESI) [37] (Figure 3).

MALDI is the most popular ionization method adaptable to MSI [38]. In MALDI, a matrix applied to the sample is excited by a laser; this energy is further transferred to the sample resulting in the ionization event [32,33]. It is particularly good at ionizing large molecules above 500 *m*/*z*, often suffering from matrix interference signals below this mass range [39]. Several groups have developed extensive work involving MALDI’s application as a platform for MSI with multiple applications into the analysis of plant samples [22]. Despite limited biologically relevant insights, these works tackle some of the main challenges in achieving comprehensive spatially resolved metabolomics, including sample preparation, the lateral resolution of ionization, and multiplex data acquisition.

Preparation for MALDI usually comprises cryo-sectioning and lyophilizing a frozen sample embedded in some media before applying the matrix by either a sprayer or solvent-free sublimation [39]. These methods offer an advantage over cell isolation in terms of metabolome integrity whilst also preserving the relative localization of cells and allowing them to assess the intercellular space [40]. However, the process still lacks significant improvements in throughput. The choice of method for matrix deposition and its composition are particularly important factors in MALDI ionization. Comparing traditional spray and solvent-free sublimation methods as an example show that the former may promote metabolite delocalization, an issue amended by the latter method in the detriment of other metabolites not being ionized [41]. Moreover, matrix crystalline structure is a relevant factor limiting lateral resolution [42]. That said, matrix optimization is an active field in technological developments for MALDI imaging applications [43]. Several works have described matrix optimization for specific compound classes [44], as well as exciting approaches to expand the coverage based on derivatizations [45] and post-ionization strategies [46]. Another recent trend involves using nanoparticles instead of organic matrixes, and it shows promising results for ionizing the smaller range of metabolites and providing increased spatial resolution [47,48].

MALDI’s lateral resolution is usually in the range of 50–10 µm, even though some reports manage to achieve numbers as low as 2–5 µm in customized systems [40,49]. Factors limiting resolution again include the matrix structure and also qualitative aspects of the laser. UV lasers provide higher resolutions of up to 10 µm. However, they have several disadvantages compared to IR lasers, such as limitations in matrix absorption [21]. SIMS is an alternative to MALDI that relies on ion beams instead of a laser to ionize the samples [50]. Such a mechanism results in a more fragmented ionization and removes the necessity of any matrix and limitations due to laser’s diffraction limit, thus providing higher reproducibility and resolution below 2 µm [50]. Moreover, SIMS allows for the acquisition of 3D imaging through the use of dual beans. Indeed, all these advantages have been recently combined in a commercial system that includes the ultra-high resolution of orbitrap analyzers [51].

Despite the advantage of more straightforward sample preparation, SIMS-based platforms’ limitation is the need for samples to be ionized under a high vacuum. A few works try overcoming such limitations, for instance, via the use of cryogenic orbiSIMS to evaluate semi-volatile organic compounds that would otherwise be vaporized before ionization [52]. Nevertheless, DESI and LAESI offer promising alternatives for direct ionization of samples with minimal treatment. In DESI, a solvent stream originated from an electrospray probe is directed at an angle toward the sample at ambient pressure, propelling secondary ions to the analyzer [53]. One of the biggest limitations of DESI is its comparatively low resolution in the order of 100 µm [50]. Finally, LAESI combines laser ablation followed by post-ionization via an electrospray. A typical resolution is in the order of 200–300 µm; however, it can reach better resolution than DESI with the additional advantage of ionizing through multiple layers of tissue [21,54,55,56].

Despite its many advantages concerning in situ analysis, MSI platforms offer significant challenges regarding data analysis [57]. As a technique in its infancy, data processing standards, such as normalization, are still lacking. Indeed, only relatively recently, an open cross-platform data format was developed [58]. Quantification is also challenging, with few works providing absolute metabolite levels [59].

## 5. Spatially Resolved Metabolomics in Plants: Current Status, Challenges, and Future Prospects

The tremendous metabolic diversity that evolved in plants at the level of tissues and organs makes single-cell metabolomics a suitable tool for investigations targeting these cell-specific chemical signatures.

To resolve metabolic diversity at a tissue level, spatial single-cell mass spectrometry, performed alone or in combination with single-cell mass spectrometry, has primarily been applied to plant science (Table 1). In *Catharanthus roseus*, for example, the combination of MSI and single-cell MS provided evidence of a developmentally driven process that segregates branches of the terpenoid indole alkaloid (TIA) biosynthetic pathway into specific anatomical structures. As idioblasts and laticifers differentiate while leaves grow and expand, single-cell MS detected the appearance of new metabolic intermediates so that an initial draft of the TIA pathway could be written in its completeness once leaves fully developed [60,61]. From an evolutionary perspective, this is a remarkable discovery as it provides an additional example of the parallel evolution between biochemical processes and anatomical structures, which often occurs in plants. In a similarly elegant experiment, Livingston and colleagues used a combination of different techniques, which included measurements of trichome intrinsic fluorescence and microcapillary-assisted metabolite extraction followed by GC-MS, UHPLC-MS/MS, and RNA sequencing analyses, to lay out the developmental trajectories of *Cannabis sativa* trichomes from sessile to stalked and the parallel changes occurring in the composition of their metabolites [62]. Questions concerning color pattern formation in flowers have also been answered via MSI. For instance, a recent study revealed that the deep-blue color of the nectar guides of *Viola cornuta* petals is due to the colocalization of the anthocyanin violanin and numerous colorless flavonol 3-*O*-glycosides [63]. By surrounding violanin, flavonol molecules prevent self-stacking and the consequent shift in the spectrum of light absorbance [64]. Additionally, they protect the chromophore of violanin from hydration, hence inhibiting the formation of colorless chalcones.

It is not accidental that the great majority of these studies focused on tissues that accumulate specialized metabolites in a large abundance and can be relatively easily accessed, such as glandular trichomes [62,65,66,67], laticifers [60,68], and floral petals [69,70,71]. Indeed, as metabolites physiologically accumulate in these organs and structures, their concentration is already optimized to detect an MS signal of sufficient quality for the molecular identification of compounds. In all other cases, to achieve a proper concentration, metabolites must be extracted from a hundred thousand identical cells. For reasons that we explain below, harvesting such a large number of cells from plant tissues is extremely labor-intensive, as it emerges when comparisons with similar systems utilized in animal studies are made.

Animal cell lines established after cell disaggregation from tissues followed by subculturing usually maintain similar physiological and biochemical characteristics as their organ of origin [72]. As such, immortal animal cell lines, for example, HeLa cells, have been successfully utilized to investigate metabolic responses to drugs and growth regulators [57]. Indeed, as metabolic changes in animal cell cultures mirror changes in intact organs, single-cell metabolomics is a powerful system to predict metabolic trajectories induced by medical treatments [73]. Conversely, plant liquid and solid (*callus*) cultures are made of cells in an undifferentiated status maintained with a balanced ratio of auxins and cytokinins [74]. As plant cell cultures are phenotypically and biochemically very distant from their differentiated counterparts, methods other than culturing must be adopted to collect a large number of cells of a specific lineage. Fluorescence-activated cell sorting (FACS) has been successfully employed to collect a large amount of GFP-tagged lines from plant tissues [27], but FACS applied to samples destined for the analysis of metabolites is a very challenging procedure. As reagents for cell protoplasting are potential contaminants of the MS detector and metabolites are prone to fast degradation, well-established methods that are used to collect RNA from fluorescent-tagged and sorted cells need further adjustments when applied to single-cell metabolomics [27]. In addition, the spatial distribution of differentiated cells in plant tissues and the prospect of obtaining protoplasts from these cells are tremendous limitations to the pursuit of harvesting cells of a single type in an amount that is sufficient for metabolite analysis. In roots, the continuous development and radial organization of layered tissues make single-cell collection via FACS relatively easily attainable, as well as facilitating the interpretation of imaging at a reasonable lateral resolution [75,76,77]. Conversely, organs that at maturity show a high degree of anatomical complexity, for example, flowers, are not equally suitable for such analyses. Not to mention that the process of protoplasting, which removes the cell wall—an intrinsic component of all plant cells— washes away compounds that cells secrete and deposit in the extracellular space. These compounds often have relevant physiological functions, for example, phenylalanine derivatives which confer protection to fungal pathogens [78]. Therefore, the removal of the cell wall can make data interpretation difficult, as it may weaken the link between chemical phenotypes and physiological functions.

These challenges are at least partially resolved when tissues undergoing single-cell analyses are already composed of a large number of identical cells. Thus, for seeds and grains where cells with well-defined chemistry spatially cluster to form seed coat, embryo, and endosperm, MSI has largely been utilized to resolve in situ localization of metabolites. For example, in the oil-seed crops *Camelina sativa* and *Brassica napus*, as well as in Arabidopsis, MSI helped to determine the distribution of lipids in the embryo of wild-type and transgenic lines [79,80,81,82,83]. In barley and wheat, the spatial distribution of sugars and proteins between the endosperm and aleurone layer has been the main object of study [84,85,86]. Furthermore, in inbreds of maize, amino acids, sugar alcohols, organic acids, phospholipids, and triacylglycerols were observed within the embryo and radicle [87]. Unicellular structures and unicellular organisms such as pollen grains, algae, and microalgae (diatoms) represent another exception. For instance, metabolomics of pollen grains, which are unicellular haploid male gametophytes, has been performed with the most disparate array of techniques. As a result of these investigations, the molecular structure and composition of sporopollenin have recently been unraveled [88,89]. Sporopollenin is an extraordinarily inert and resistant polymer, the acquisition of which by land plants represents a focal adaptation to life outside water. The spectacular inertness of sporopollenin toward the most disparate analytical techniques made the search for its molecular structure hard to obtain, and at the same time, very desirable given the multitude of promising applications in the fields of material engineering and nanotechnology. Progress has also been made in the analysis of lipids, proteins, and the mechanisms of accumulation of flavonoid glycosides on the surface of pollen grains [90,91,92,93], as well as the metabolic processes underlying pollen germination and pollen tube elongation [94]. However, given the complexity of these chemical signals, their physiological function is not yet fully understood [95]. Finally, we briefly mention here that while collecting abundant pollen from male microsporangia of gymnosperms is usually easy endeavors, autogamous angiosperms generally produce a tiny amount of pollen, for which the collection of whole anthers is a necessary step.

In algal research, live single-cell metabolomics helped elucidate the metabolic rearrangements occurring in response to environmental perturbations such as low nutrient and variation in light regimes [96,97,98]. Phytoplankton, which is primarily composed of microalgae and minor amounts of protists and bacteria, contributes to global biogeochemical cycles of carbon, nitrogen, phosphorus, and silicate. Therefore, gaining an understanding of the physiological status of phytoplankton cells holds excellent promises for environmental research. Besides, chemotyping of microalgae via pipelines that utilize live single-cell MS is currently exploited for taxonomic identification [99]. Despite the broad applications in environmental research, initial studies on algal metabolomics mostly focused on the model organism *Chlamydomonas reinhardtii* because of applications in the biotechnology industry and biofuel production [100]. In the recent past, metabolomic and transcriptomic approaches have been extensively utilized to investigate the responses to external determinants of algal growth such as temperature, light intensity, salinity, and nutrient availability [101,102], while today’s research mostly shifted toward functional genomic studies that aim at understanding the genetic mechanisms of this metabolic plasticity [103].

In plants, the application of single-cell MS to functional genomic studies has so far been scant, although initial studies that employed known *Arabidopsis thaliana* mutants as a proof of concept showed great promises [104,105,106]. More recently, the combination of direct infusion metabolomics and MSI was used to characterize the signaling pathway of *feronia* mutants, revealing an interesting phenotype associated with high levels of oxylipin arabidopsides, and suggesting chloroplastic localization [107]. Other new prospects of single-cell metabolomic applications pertain to research on plant–pathogen interactions where MSI is currently being utilized to analyze plant metabolites synthesized in response to pathogens’ infection. Here, initial studies that used to visualize metabolites present on the plant surface have further expanded to include analysis of metabolites that accumulate deeper in plant tissues, which can be seen after tissue fracturing and sectioning [108]. A recent study has shown this new approach’s relevance when it analyzed transcriptome and metabolome responses of susceptible and resistant soybean cultivars to aphid infestation. As aphids are insects that feed on phloem sap, plant metabolites conferring resistance to aphids are expected to be found in the phloem. Conversely, MSI revealed the accumulation of isoflavones in mesophyll and epidermal cells, suggesting a role for these compounds in the non-phloem defense response induced by feeding [109]. MSI has also been used to investigate the distribution of glucosinolates across Arabidopsis leaves and the response of lepidopterans oviposition to the detected metabolites’ concentrations [59]. In plant–bacteria symbiotic associations, MSI has been employed to study metabolite distribution in roots and nodules of wild-type and mutant genotypes of *Medicago truncatula* [110,111], and more recently of soybean [112,113].

Still, single-cell metabolomics has found very little application in the field of plant developmental research where the combination of functional genomics and metabolomics holds the promise to pave the way toward a better understanding of how and to what extent anatomy and metabolism are mutually coordinated. This is at least partially due to challenges related to sample preparation, as the presence of abundant water, cell wall, and cuticles make the process laborious in plants [114]. Additionally, the resolution and annotation of metabolites of the central pathway, which accumulate in lower abundance than specialized metabolites (see above), represent an additional challenge. Similarly, phytohormones that play crucial roles at the cellular level in the development and environmental responses are of great interest but challenging to detect even with traditional methods [115]. A work using the “Live-MS” platform [116] to investigate the response of two phytohormones, ABA and JA-Ile, have shown promising results being able to detect some of the expected changes. However, their results also point to limitations of the technique which still suffers from high variability. Finally, it is worth mentioning that plants primarily utilize hexoses and various sugar polymers for storage, transport, and organ to organ communication, of which MS annotation is not always easily attainable.

**Table 1:** Summary of spatially resolved plant metabolomics works applying mass spectrometry-based platforms described in this review.

Species	Technique	Cell-Type/Tissue	Compounds	Reference
Arabidopsis	FACS	Roots	Multiple	[27]
Arabidopsis	MALDI	Leaves	Glucosinolates	[60]
*Catharanthus roseus*	MALDI and Live-MS	Laticifers and idioblasts from leaves	TIA	[61]
*Catharanthus roseus*	MALDI and Live-MS	Laticifers, idioblast, parenchyma, and epidermal cells from stems	TIA	[62]
*Viola cornuta*	MALDI	Petals	Flavonoids	[64]
*Rauvolfia tetraphylla*	DESI	Stem, leaves, root, and fruits	Indole alkaloids	[69]
*Hypericum perforatum*	DESI	Petals and leaves	Hyperforin	[71]
*Datura stramonium*	DESI	Petals and leaves	Sugars, atropine, and scopolamine	[71]
Maize	MALDI	Roots	Amino acids	[76]
Maize	MALDI	Roots	Lipids, sugars, and benzoxazinoid	[77]
*Glycyrrhiza glabra*	MALDI	Roots	Flavonoids and triterpenoids	[78]
*Camelina sativa*	MALDI	Seed	Lipids	[80]
*Camelina sativa*	MALDI	Seed	Lipids	[81]
*Camelina sativa*	MALDI	Seed	Lipids	[82]
*Brassica napus*	MALDI	Seed	Lipids	[83]
Arabidopsis	MALDI	Seed	Lipids	[84]
Barley	MALDI	Germinating seeds	Multiple	[85]
Maize	MALDI	Germinating seeds	Multiple	[88]
*Lycopodium clavatum*	SIMS and MALDI	Polen	Sporopollenin	[90]
*Poa alpina*	MALDI	Polen	Multiple	[91]
Arabidopsis	MALDI	Leaves	Oxylipins	[108]
Rice	MALDI	Leaves	Multiple	[109]
Soybean	MALDI	Leaves	Multiple	[109]
Soybean	MALDI	Leaves	Isoflavones	[110]
*Medicago truncatula*	MALDI	Root nodules	Multiple	[111]
*Medicago truncatula*	MALDI	Root nodules	Multiple	[112]
Soybean	MALDI	Root nodules	Multiple	[113]
Soybean	LAESI	Root nodules	Multiple	[114]
*Vicia faba*	Live-MS	Leaves	Phytohormones	[117]

## 6. Conclusions and Future Perspectives

There are just over 40 different types of cells described in plant tissues [117]. As most metabolomics experiments capture data of whole tissues, our knowledge is largely biased toward prevailing cells such as mesophyll cells in leaves [118] and endosperm in seeds [119,120]. However, several works highlight the striking differences in cell-specific metabolism and the impact that less recurrent cell types have in regulating and integrating crucial physiological processes, including transpiration and photosynthesis [121,122]. Moreover, assessing metabolic heterogeneity across cells belonging to a tissue has the potential to unravel unforeseen details masked by averaging such populations of cells, thereby contributing to a deeper understanding of metabolic regulation [6].

Techniques for measuring single-cell metabolites have recently gone through considerable improvements providing exciting insights into metabolic compartmentalization. Nevertheless, some of the metabolomics most outstanding achievements rely on high throughput and comprehensive metabolome coverage. Both parameters are still considerably limited in the current single-cell and spatially resolved platforms. The many advantages of single-cell profiling described here represent an enormous potential when applied to large throughput experiments. Single-cell transcriptomics of different human tissues has recently been utilized to identify Quantitative Trait Loci (QTL) associated with expression and splicing variants (eQTLs and sQTLs, respectively) affected by the background genetic variation of different individuals [123]. A similar approach to plant tissues has not yet been adopted. However, it represents a significant potential if applied to large populations to understand, among others, the effect of environmental perturbations at a single-cell level.

Improvements in various aspects of mass spectrometry aspects, particularly resolution and sensitivity, have been instrumental in facilitating the measurement of the spatial distribution of metabolites through single-cell and MSI platforms. The introduction and broad adoption of other technologies into metabolomics platforms, such as nanoLC and ion mobility, are likely to play important roles in further reducing issues concerning limited sample and sensitivity in single-cell metabolomics and matrix effects in MSI, respectively. Better integration of current technologies with other imaging platforms such as microscopy also offers a promising way to improve experiments throughput and information [73].

Finally, as these technologies mature, we can foresee their adoption to even the most challenging applications in current tissue level metabolomics. A recurrent question that has proven essential to characterize metabolism is the definition of metabolic fluxes rather than a simple description of relative metabolite levels as routinely performed [124]. Methods for integrating multi-omics of single cells are also an exciting boundary to be crossed [125]. We can anticipate considerable hurdles for generating such datasets. However, this could represent an outstanding means of reducing experimental complexity while improving the statistical power of systems biology studies.

## Abbreviations

DESI	Desorption electrospray ionization
FACS	Fluorescence-activated cell sorting
GC	Gas chromatography
LAESI	Laser-ablation electrospray ionization
LC	Liquid chromatography
LCM	Laser capture microdissection
LMD	Laser microdissection
LMPC	Laser microdissection and pressure catapulting
MALDI	Matrix-assisted laser desorption/ionization
MS	Mass spectrometry
MSI	Mass spectrometry imaging
QTOF	Quadrupole time-of-flight mass spectrometer
SIMS	Secondary ion mass spectrometry
TIA	Terpenoid indole alkaloid
UHPLC	Ultra-high-performance liquid chromatography
UV	Ultraviolet
